# Neighborhood Social Environment and Self-Perceptions of Aging

**DOI:** 10.1093/geroni/igad038

**Published:** 2023-04-27

**Authors:** Eun Young Choi, Elizabeth M Zelinski, Jennifer Ailshire

**Affiliations:** School of Global Public Health, New York University, New York, New York, USA; Leonard Davis School of Gerontology, University of Southern California, Los Angeles, California, USA; Leonard Davis School of Gerontology, University of Southern California, Los Angeles, California, USA; Leonard Davis School of Gerontology, University of Southern California, Los Angeles, California, USA

**Keywords:** Attitudes toward own aging, Ecological model, Neighborhood social cohesion

## Abstract

**Background and Objectives:**

Self-perceptions of aging (SPA) are associated with health and well-being later in life. Although prior studies have identified individual-level predictors of SPA, the role of neighborhood social context in SPA remains largely unexplored. A neighborhood social environment may act as a critical avenue for older adults to remain healthy and socially active, contributing to their evaluations of how they grow old. The present study aims to fill the previous research gap by examining the relationship between neighborhood social environment and SPA, and how age may moderate this relationship. This study is guided by Bronfenbrenner’s Ecology of Human Development theory and Lawton’s Ecological Model of Aging, positing that an individual’s aging experience is deeply rooted in their residential environment.

**Research Design and Methods:**

Our sample includes 11,145 adults aged 50+ from the 2014 and 2016 waves of the Health and Retirement Study. We included 4 social and economic aspects of neighborhoods: (1) neighborhood poverty; (2) percentage of older adults; (3) perceived social cohesion; and (4) perceived disorder.

**Results:**

Multilevel linear regression models showed that respondents in neighborhoods with higher percentages of the older population and with perceptions of high neighborhood disorder reported more negative SPA. Those who perceived their neighborhoods as more socially cohesive reported more positive SPA. Controlling for individual socioeconomic and health status, only neighborhood social cohesion remained significant. We also found significant interaction effects between neighborhood social cohesion and age: The effects of neighborhood cohesion on SPA were stronger in middle age than in old age.

**Discussion and Implications:**

Our findings provide insights into how neighborhood social context is associated with SPA, suggesting that a socially cohesive neighborhood may be important to promote more favorable perceptions of aging, particularly for middle-aged residents.

**Translational Significance:** This study found that older adults perceiving neighborhoods to be highly socially cohesive had more positive self-perceptions of aging. Our focus on neighborhood social contexts provides some of the first insights into how one’s reflections and evaluations of their own aging are embedded in their living environment, suggesting that a socially cohesive neighborhood may be critical to promoting more favorable perceptions of aging, particularly for middle-aged residents. Extending the prior focus on individual-level interventions, our study emphasizes the need for community intervention strategies to bolster the cohesion of neighborhoods, which can translate into better self-perceptions of aging among older residents.

## Background and Objectives

The term “self-perceptions of aging (SPA)” refers to individuals’ evaluations of their own aging process (Levy, 2003), such as feeling more useless and thinking things get worse as they age. SPA is conceived as a fundamental process of adult development and constitutes an integral part of the intentional self-development (Diehl et al., 2015). Negative SPA is associated with older adults’ greater disease burden, poor physical and mental health, impaired functional ability, and even mortality risk, as well as lower levels of well-being and higher levels of social isolation (Chang et al., 2020, for reviews). In recognition of its importance, previous literature has identified individual-level correlates of SPA such as education, health status, and experience of age discrimination (Kornadt et al., 2021). However, the attempts to link it to broader contextual-level factors have been limited (Marques et al., 2020). Addressing the impacts of macro-level contexts, such as cultural, societal, and residential environment, would lead to a better understanding of the formation of SPA.

The current research is guided by two ecological frameworks: Bronfenbrenner’s Ecology of Human Development theory (1977) and Lawton’s Ecological Model of Aging (1973). These frameworks are particularly relevant as they consider not only social contexts, such as social relations, which have been a longstanding focus in social gerontology, but also the role of the immediate physical environment, such as features of the home and community, which have been largely neglected in other conceptual frameworks (Wahl, 2015). Specifically, we will evaluate the role of neighborhood social environment as an integral context where individuals develop SPA through the interaction between the residential environment and psychological processes. Neighborhood environments may serve as a critical avenue for older individuals to remain healthy and socially active through socioeconomic and interpersonal resources, which in turn contributes to their evaluations of growing old. About 98% of Americans aged 65 and older live in the community (Barkoff, 2021), and the absolute number is projected to increase with population aging. Moreover, older adults aged 65 and above spend most of their daytime hours in their community (Oswald & Wahl, 2005) and have the greatest levels of socialization with neighbors compared to other age groups (Cornwell et al., 2008). In this study, we focused on individuals aged 50 or above, a broader definition of older adults, as it is the earliest cutoff that has been often adopted in prior studies on SPA (Tully-Wilson et al., 2021) and also allows for exploring age interactions in the association between neighborhoods and SPA. Knowledge about how one’s reflections and evaluations of their own aging are embedded in their living environment will not only contribute to the literature on SPA, but also elucidate the psychological mechanisms through which neighborhoods influence older adults’ health, which is another area that has been underexplored (Yen et al., 2009).

### Theoretical Backgrounds

The foundations of SPA are grounded within the self, incorporating aspects of one’s self-concept. People need a sense of self to be able to evaluate whether their aging process is positive or negative. In this sense, the environmental psychology studies of the self provide theoretical support for the potential association of neighborhood social environments with SPA. As early as the 1890s, psychological theories suggested that the self is intertwined with the outside world (James, 1890), conceptualizing the self as a product of one’s reflections from other people and symbols existing in one’s social environment (Mead, 1934). As such, places have been regarded as driving sources for the development of self-identity as they embody social symbols and provide an affective bond for residents (Proshansky, 1978). In addition, individuals establish personal meaning by locating themselves within their residential communities throughout daily routines and during exceptional circumstances, from which places constitute a symbolic extension of the self (Pretty et al., 2003). Old age, in particular, has been seen as an important period of the life span where local environments have increasing significance in relation to the self-identity (Bonnes et al., 2003). This body of literature suggests that SPA may be constructed, developed, and changed by broader environmental contexts, rather than a product of solely individual-level factors.

The Ecology of Human Development framework assumes that human development occurs through being embedded within multiple layers of the external environments, from immediate built and social settings to broad cultural values and customs (Bronfenbrenner, 1977). In the field of gerontology, the Ecological Model of Aging (Lawton & Nahemow, 1973) similarly orients attention to how physical and social aspects of the neighborhood context influence individuals’ aging process over time, thereby contributing to aging well (Wahl et al., 2012). Both perspectives suggest that one’s aging experience is structurally embedded in the residential environment—where they live and what resources they have within a community. The subsequent consequences of living in a disadvantaged neighborhood environment can include (a) producing or reinforcing ageism, (b) increasing the risk for isolation in later life, and (c) impeding the health and emotional well-being of older residents (Hagestad & Uhlenerg, 2005). Likewise, individuals’ evaluations of their aging are likely to be influenced by personal daily experiences and interactions with others in their residential communities (Miche et al., 2014).

The neighborhood social environment broadly refers to the sociodemographic composition of the neighborhood, as well as the relationships and social processes among residents living in a certain neighborhood (Cagney et al., 2009). Researchers have operationalized and measured neighborhood social environments in two ways. The first group uses sociodemographic data as indicators of neighborhood socioeconomic status, including poverty rates (Subramanian et al., 2006) and the percentage of older adults for geographically defined areas such as census tracts (Stokes & Moorman, 2016). The second group focuses on subjective perceptions of neighborhood physical and social elements by using participants’ responses for their feelings about residential areas, or everywhere within a 20-min walk (Yen et al., 2009). Research supports that neighborhood social environment, either measured with objective indicators or self-report, has been an important determinant of older adults’ health and well-being. For example, neighborhood socioeconomic disadvantage and low perceived neighborhood support are associated with a variety of poor health conditions, including higher levels of depressive symptoms (Kubzansky et al., 2005) and morbidity (Nordstrom et al., 2004). Although this body of theoretical frameworks and empirical evidence suggests the critical role of contextual-level factors in aging in general, a dearth of empirical research has identified the neighborhood predictors of how one perceives their own aging process. Only a couple of studies investigated the relationship of regional characteristics with SPA so far (Wolff et al., 2018; Wurm et al., 2014), where older adults living in districts with fewer economic resources (low GDP) and a faster population aging rate reported more negative SPA. However, these studies primarily come from the sample of older adults in Germany and included a limited selection of indicators of neighborhood environment, which calls for further investigation.

It is important to note that neighborhood social environment may not have equal effects across age groups. There is a great diversity among people aged 50 and above. As one of the first attempts, Neugarten (1975) argued for a heterogeneous view of older adults by suggesting distinct age categories of the young-old (55–75) and the old-old (75+). During the transition from mid-life to older adulthood, many individuals experience dramatic changes in work (e.g., retirement), social relationships (e.g., bereavement), and health (e.g., development of diseases; Skerrett et al., 2021), which may drive substantive changes in SPA (Chopik et al., 2018). In addition, these differences between the two groups with regard to health, work, and leisure are likely to result in different needs in what they want for their local neighborhood environment. Middle-aged adults may look more for community engagement or continuing education opportunities to maximize their leisure time following retirement. Relatively healthy but retired adults in their 60s are likely to have positive SPA but may endorse more negative SPA if they live in areas with a lack of supportive services needed for social engagement. In contrast, older adults aged 75 or above, who on average have more chronic conditions than the middle aged, would need special features or health services in neighborhoods to enable them to remain as fully functional as possible. In this sense, age groups are likely to differ in terms of which aspects of the neighborhood are significantly associated with SPA and the strength of the associations.

### Present Study

Elucidating the role of the environmental context in which we live will provide a more complete picture of the factors associated with SPA. To this end, the present study aims to determine whether the neighborhood sociodemographic environment and perceived neighborhood characteristics are associated with SPA, controlling for individual-level socioeconomic and health status. Drawing from the existing literature on neighborhood environments and aging outcomes discussed above (Subramanian et al., 2006; Wolff et al., 2018; Yen et al., 2009), we focused on four aspects of the neighborhood social environment: (1) poverty rates as an indicator of neighborhood socioeconomic conditions; (2) proportions of adults aged 65 or above as an indicator of neighborhood-level age profiles; (3) perceived neighborhood social cohesion as an indicator of mutual trust and solidarity among neighbors; and (4) perceived neighborhood disorder as an indicator of breakdown of order and social control. We hypothesized that poor neighborhood social conditions would be associated with more negative SPA (**Hypothesis 1**). The secondary aim of the present study is to investigate the age differences in the associations of the neighborhood social environment with SPA. We hypothesized that there would be significant interactions between ages and the indicators of neighborhood social environment on SPA (**Hypothesis 2**). Given the dearth of existing knowledge, we will maintain an exploratory approach for this part of the investigation and not specify directions for the associations.

## Research Design and Methods

### Data and Sample

This study uses data from the Health and Retirement Study (HRS). The HRS is a longitudinal survey of a U.S. nationally representative sample of individuals aged 50 years and older, and it has been conducted biennially since 1992. The HRS is approved by the University of Michigan’s Institutional Review Board. A wide range of information on sociodemographic characteristics and health status has been collected through face-to-face or telephone interviews. In 2006, a random half of the noninstitutionalized sample was administered the Psychosocial and Lifestyle Self-Administered Questionnaire (SAQ), and the other half received the SAQ in 2008, with the design repeated every 4 years. The current study derived data from the 2014 and 2016 HRS surveys, when questions on neighborhood social capital (relatives and friends) were added. A total of 6,774 and 5,165 community-dwelling adults aged 50 and above completed the questionnaire in 2014 and 2016, respectively. Cases with missing study variables were excluded (*n* = 794), resulting in the final analytic sample of 11,145. The neighborhood poverty rates and the percentage of older adults were obtained from the HRS Contextual Data Resource, which was constructed using U.S. Census Bureau’s 2012–2016 American Community Survey 5-year estimates (Ailshire et al., 2020). This study linked the census-tract-level neighborhood information to HRS respondents with census-tract identifiers.

### Measures

#### Poverty rates and proportion of older adults

The percent of residents with income below the federal poverty threshold was calculated for each census tract. Then, census tracts were categorized into three groups based on Crane (1991): (1) neighborhoods with low poverty rates: 0%–19.99%; (2) moderate poverty: 20%–39.99%; (3) high poverty: 40% or higher. The percent of older residents aged 65 or above among the total population was calculated for each census tract. The population counts included individuals in group quarters. Following the Organisation for Economic Co-operation and Development (OECD) and World Health Organization guidelines (2020), census tracts were grouped into three categories: (1) nonaging neighborhoods: less than 7%; (2) aging neighborhoods: 7%–13.99%; (3) aged neighborhoods: 14%–20.99%; and (4) super-aged neighborhoods: 21% or higher.

#### Perceived neighborhood social cohesion and neighborhood disorder

Respondents were asked to report their feelings about one’s local area, or everywhere within a 20-min walk or about a mile of one’s home. Responses were coded on a 7-point Likert scale (1 = *most disagreement*; 7 = *most agreement*). The measure (Cagney et al., 2009) included four items for social cohesion (e.g., I feel part of this area), and four items for physical disorder (e.g., vandalism/graffiti is a big problem in this area). The average scores were calculated (range: 1–7), with higher scores indicating higher levels of cohesion (Cronbach’s α = 0.86 in 2014 and 0.87 in 2016) and higher levels of disorder (Cronbach’s α = 0.84 in 2014 and 0.85 in 2016). Those with more than two missing items were considered as missing on the final score for each index. This study created three categories of low (mean values ≤3), middle (mean values >3 and ≤5), and high (mean values >5) for both cohesion and disorder indicators.

#### Self-perceptions of aging

In the HRS, SPA was measured using eight items. Five questions were based on the subscale of the Philadelphia Geriatric Center Morale Scale (Lawton, 1975): (1) “Things keep getting worse as I get older,” (2) “I have as much pep as I did last year,” (3) “The older I get, the more useless I feel,” (4) “I am as happy now as I was when I was younger,” and (5) “As I get older, things are better than I thought they would be.” An additional three items were derived from the Berlin Aging Study (Kotter-Grühn et al., 2009): (6) “So far, I am satisfied with the way I am aging,” (7) “The older I get, the more I have had to stop doing things that I like,” and (8) “Getting older has brought with it many things that I do not like.” An additional three items from the Berlin Aging Study were included to increase reliability for a unidimensional scale (Smith et al., 2017). Participants rated each question on a 6-point Likert scale (1 = *strongly disagree*; 6 = *strongly agree*). After reverse coding items (1), (3), (7), and (8), the scores across eight items were averaged, with a higher score indicating more positive perceptions. The psychometrics of the scale had good internal consistency (Cronbach’s α = 0.81 in 2014 and 0.81 in 2016).

#### Covariates

In line with prior research (Marques et al., 2020), we included individual-level demographic factors, socioeconomic status, and health conditions as control variables in our analysis, to account for their known impact on SPA. We used demographic information from the 2014 wave for the sample in 2014 and information from the 2016 wave for the sample in 2016. Demographic factors included age, gender (1 = female), race/ethnicity (1 = non-Hispanic White; 2 = non-Hispanic Black; 3 = Hispanic; and 4 = others) and living arrangement (1 = married or partnered; 2 = single, living with others; 3 = single, living alone). Education in years and total household income were used as indicators of socioeconomic status. Education was assessed with years of formal schooling (range: 0–17), and household income was measured by income from all possible sources such as earnings, pensions, and social security. Due to high skewness, log-transformed values of income were used for the multivariate analyses.

With regard to physical health conditions, this study considered the number of chronic diseases and functional limitations at each wave. The respondents reported whether they had any of the following eight chronic diseases diagnosed by a physician (high blood pressure, diabetes, cancer, lung disease, heart disease, stroke, psychiatric problems, and arthritis); then, the sum value was calculated (range 0–8). For the functional limitations, the respondents indicated whether they had any limitations in activities of daily living, including bathing, dressing, walking across a room, and getting in and out of bed. Summary scores were calculated (range: 0–5) and a new binary variable (1 = having any functional limitations) was created for the multivariate analyses. As a mental health indicator, depressive symptoms were assessed by the eight-item Center for Epidemiological Studies—Depression (CES-D) scale, a shortened version of the original CES-D scale (Radloff, 1977). The respondents were asked to report whether they experienced the following sentiments most or all of the time: (1) felt depressed, (2) everything is an effort, (3) sleep is restless, (4) felt alone, (5) felt sad, (6) could not get going, (7) felt happy, and (8) enjoyed life. Total scores were calculated by subtracting the two positive indicators from the negative items, with a possible range of 0–8. Internal reliability was acceptable, with the Kuder–Richardson 20 (KR-20) coefficient of 0.80. Finally, we also controlled individuals’ neighborhood social capital. Respondents were asked to report whether they had any good friends living in their neighborhood. A binary variable was created for neighborhood friends (1 = *yes*). Respondents were asked to report whether they had any relatives (besides people living with them) in their neighborhood. Another binary variable was created for neighborhood relatives (1 = *yes*).

### Analytical Strategy

The descriptive characteristics of the study variables were reviewed. Next, multilevel linear regression models were estimated to explore the associations between neighborhood social environment factors and SPA. After checking the intraclass correlation (ICC) in the unconditional random intercept model, two nested models were examined: (1) neighborhood characteristics at the census-tract level (i.e., poverty rates and proportion of older adults) and individual level (perceived neighborhood social cohesion and neighborhood disorder) were included controlling for demographic factors, and (2) socioeconomic and health status were further adjusted to test whether they explain the observed neighborhood characteristics and SPA relationship. Sample weights were applied to adjust for survey nonresponse and oversampling of African American and Hispanic populations. The level of statistical significance was set at a two-tailed alpha level of 0.05 for all analyses. All analyses were performed using Stata 17.0.

## Results

### Sample Characteristics

Table 1 presents the descriptive statistics of the sample. The mean age of the participants was 66.4 years, about 54% were female. The majority of the sample consisted of non-Hispanic White (77.5%), followed by non-Hispanic Black (10.1%), Hispanic (8.6%), and other racial/ethnic minorities (3.8%). About 67% were married or partnered, and among singles, 11% lived with others (either family members or nonrelated others), and 22% lived alone. On average, the participants had 13 years of education, 87K dollars of annual household income, and about two chronic diseases. The scores of functional limitations and depressive symptoms averaged 0.3 and 1.3, with about 15% reporting one or more functional limitations.

**Table 1.** Descriptive Sample Characteristics

Variable	*M* ± *SD*	%
Self-perceptions of aging (range: 1–6)	3.91 ± 1.06	
Neighborhood social environment		
Sociodemographic condition		
Poverty	14.65 ± 0.11	
Low poverty (range: 0–19.9)		75.5
Moderate poverty (range: 20–39.9)		20.2
High poverty (range: 40–75)		4.1
Older population aged 65+ (%)	16.36 ± 0.16	
Nonaging neighborhood (range: 0–6.9)		5.5
Aging neighborhood (range: 7–13.9)		37.0
Aged neighborhood (range: 14–20.9)		40.4
Super-aged neighborhood (range: 21–87.2)		17.1
Subjective perceptions measured by self-report		
Perceived social cohesion (range: 1–7)	5.37 ± 1.37	
Low cohesion (range: ≤3)		8.3
Middle cohesion (range: >3 and ≤5)		26.7
High cohesion (range: >5)		65.1
Perceived disorder (range: 1–7)	2.48 ± 1.41	
Low disorder (range: ≤3)		73.5
Middle disorder (range: >3 and ≤5)		19.8
High disorder (range: >5)		6.7
Demographics		
Age (range: 50–98)	66.36 ± 9.95	
Gender		
Male		46.0
Female		54.0
Race/ethnicity		
Non-Hispanic White		77.5
Non-Hispanic Black		10.1
Hispanic		8.6
Other		3.8
Living arrangement		
Married or partnered		66.6
Single, living with others		11.1
Single, living alone		22.3
Social capital in neighborhood		
Having good friends		42.9
Having relatives (not co-living)		23.2
Socioeconomic factors		
Education (in years)	13.34 ± 2.93	
Annual household income (USD)	86,849 ± 130,555	
Health conditions		
Chronic diseases (range: 0–8)	2.14 ± 1.51	
Functional limitations (range: 0–5)	0.29 ± 0.82	
Having any limitations		14.8
Depressive symptoms (range: 0–8)	1.32 ± 1.92	

*Notes*: Racial/ethnic identification of “other” included American Indian, Alaskan Native, Asian, and Pacific Islander. *M* = Mean; *SD* = standard deviation.

The overall mean score of the SPA measure was 3.9 on a 6-point scale, indicating a slightly skewed distribution toward the positive perception. Regarding the neighborhood sociodemographic environment, the average poverty rate was 14.7% and 16.4% for the population aged 65+. The average perceived neighborhood social cohesion was moderately high, about 5.4 on a 7-point scale, and the mean level of perceived neighborhood disorder was below the midpoint (mean = 2.5). On average, respondents’ perceptions of their neighborhood were favorable. About 43% had good neighborhood friends, and 23% reported having relatives (not co-living) in their neighborhood.

### Neighborhood Social Environment and SPA

Table 2 presents the results of the multilevel linear regression models predicting SPA. In the unconditional random intercept model without any predictors, we found ICC of 0.16 at the census-tract level. These numbers represent the correlation in SPA between two older adults within the same census tract. We can also conclude that 16% of the variation in SPA can be attributed to the census tract. Unadjusted Model tested the associations of the neighborhood social environment indicators with SPA. Respondents who lived in the neighborhoods with higher percentages of the older population reported more negative SPA. On the other hand, those who perceived their neighborhoods as more socially cohesive and having less disorder reported more positive SPA. After controlling for demographic, socioeconomic, and health status (Adjusted Model), only the perceived neighborhood social cohesion remained significant. The significance of % of the population aged 65+ disappeared when adjusting for age, and the significance of perceived disorder disappeared when adjusting for socioeconomic or physical health status.

**Table 2.** Multilevel Linear Regression Models Estimating Self-Perceptions of Aging

Variable	Unadjusted model		Adjusted model	
	*B*	*SE*	*B*	*SE*
*Neighborhood social environment*				
Sociodemographic condition				
Poverty (Ref = low poverty rates)				
Moderate poverty	−0.04	0.03	0.00	0.02
High poverty	−0.01	0.05	0.01	0.05
% Population aged 65+ (Ref = nonaging)				
Aging neighborhood	-0.04	0.05	0.03	0.04
Aged neighborhood	−0.11*	0.05	0.01	0.04
Super-aged neighborhood	−0.14*	0.05	0.00	0.05
Subjective indicator				
Perceived social cohesion (Ref = low)				
Middle	0.02	0.05	−0.04	0.04
High	0.40***	0.05	0.24***	0.05
Perceived disorder (Ref = low)				
Middle	−0.08*	0.03	−0.04	0.03
High	−0.11*	0.05	−0.03	0.05
*Covariates*				
Age			−0.01***	0.00
Female			0.10***	0.02
Race/ethnicity (Ref = non-Hispanic White)				
Non-Hispanic Black			0.34***	0.03
Hispanic			0.23***	0.04
Other			0.02	0.05
Living arrangement (Ref = married/partnered)				
Single, living with others			0.01	0.03
Single, living alone			0.04	0.02
Individuals’ social capital in neighborhood				
Having good friends			0.06***	0.02
Having relatives (not co-living)			0.01	0.02
Years of education			0.02***	0.00
Annual household income (log-transformed)			0.04***	0.01
Having functional limitations			−0.36***	0.03
Number of chronic diseases			−0.11***	0.01
Depressive symptoms			−0.17***	0.01
Intercept	3.74***	0.07	4.21***	0.13
Random effects	Variance	*SE*	Variance	*SE*
Level 2 (census tract)	0.16***	0.01	0.06***	0.01
Level 1 (individuals)	0.90***	0.02	0.71***	0.12

*Notes*: Unadjusted Model only included neighborhood indicators; Adjusted Model controlled for individual-level demographics, socioeconomic status, health conditions, and social capital in neighborhood. Ref = reference; *SE* = standard error.

* *p* < .05. *** *p* < .001.

### Age Differences in the Effects of Neighborhood Social Environment on SPA

**Figure 1.**
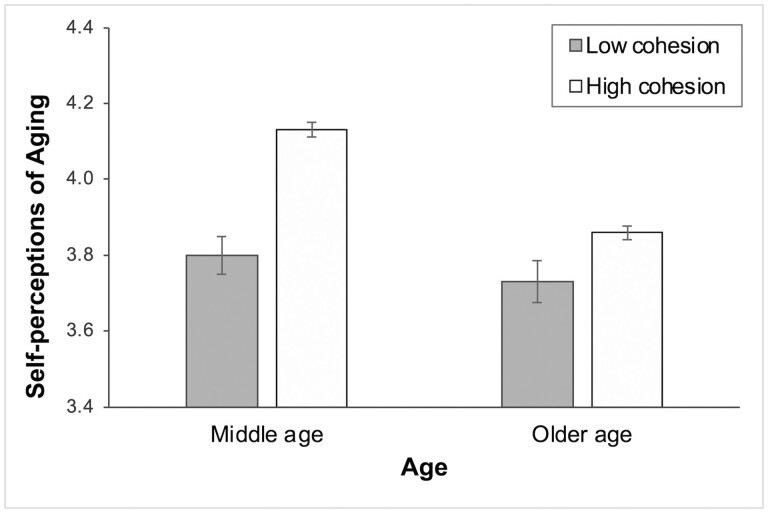
Age differences in the effects of social cohesion on self-perceptions of aging. Middle age was about 56 years old (−1 *SD*); older age was 76 years old (+1 *SD*). *SD* = standard deviation.

Using the same modeling approach, we next tested whether the strength of the associations between neighborhood social environment indicators on SPA interacted with age. Table 3 presents statistically significant interaction effects between age and social cohesion after controlling for all other covariates (*B* = −0.01, standard error = 0.003, *p* < .01). The effects of neighborhood cohesion on SPA were stronger in middle ages than in older ages (age was a continuous variable in the model). However, no significant age differences were observed in the effects of other neighborhood indicators (results not shown, available upon request). To further explore the significant interaction effects, we plotted the predicted SPA scores by age categories and conducted post-hoc pairwise comparisons. As shown in Figure 1, middle-aged respondents (−1 *SD* [standard deviation], about 56 years old) living in the low cohesion neighborhoods reported comparable SPA scores to older respondents (+1 *SD*, about 76 years old) in neighborhoods with high cohesion. The gaps between low and high neighborhood social cohesion were larger in middle ages than in older ages, indicating the stronger effects of neighborhood social cohesion on SPA among middle-aged respondents.

**Table 3.** Multilevel Linear Regression Models Estimating Interaction Effects of Perceived Neighborhood Social Cohesion and Age

Variable	*B*	*SE*
Perceived neighborhood social cohesion (Ref = low) × Age		
Middle × Age	−0.01	0.004
High × Age	−0.01**	0.003
Perceived neighborhood social cohesion (Ref = low)		
Middle	−0.05	0.04
High	0.22***	0.05
Age	−0.003	0.003

*Notes*: All other covariates (individual-level demographics, socioeconomic status, health conditions, and social capital in neighborhood) were controlled, but not shown for simplicity. *SE* = standard error.

** *p* <.01; *** *p* <.001.

## Discussion and Implications

Despite the importance of SPA for health and well-being in later life, very little is known about the role of contextual-level factors. Research has documented the increased focus on socially and emotionally meaningful goals in later life (Carstensen et al., 1999). Several studies have shown that the neighborhood environment plays a crucial role in achieving these goals, as a socially cohesive neighborhood can foster supportive relationships and a sense of belonging, thereby promoting well-being among older adults (Cramm & Nieboer, 2015). Our study adds to this literature by examining how SPA among older residents can be related to their neighborhood social environment. Specifically, two research hypotheses were tested: (1) whether a poor neighborhood sociodemographic environment is associated with more negative SPA and (2) whether age moderates the associations of significant neighborhood indicators with SPA. Four social and economic indicators of poor neighborhoods were considered: (1) high neighborhood poverty; (2) high density of older adults; (3) perceived low social cohesion; and (4) perceived high physical disorder.

The results provided partial positive support for the first hypothesis and the second hypothesis. Respondents in the neighborhood with more older populations reported more negative SPA. On the other hand, those who perceived their neighborhoods as more socially cohesive and having less disorder reported more positive SPA. Individual demographic, socioeconomic, and health status explained some of these associations for percent of older population and perceived neighborhood disorder, but the perceived neighborhood social cohesion remained a significant predictor of SPA after the adjustment. These findings are in line with the broader literature on the association of neighborhood environment with self-concepts, which showed that neighborhood deprivation (e.g., high poverty and unemployment) was associated with poor self-efficacy and self-worth (Boardman & Robert, 2000; Fagg et al., 2013). In addition, there were significant age differences in the effects of neighborhood social cohesion on SPA, but not other neighborhood characteristics, such that the main effects of perceived neighborhood cohesion were stronger in a middle age group than those over the age of 75.

Our findings suggest that perceived social connection aspects of neighborhood environment are most closely associated with SPA among older adults, independent of individuals’ characteristics and other socioeconomic conditions of neighborhoods. Notably, social cohesion was a consistent factor of SPA across models, even controlling for individuals’ neighborhood social networks and living arrangements. Neighborhood social cohesion characterizes social resources available within the whole community and thus is distinct from individual-level social capital and support (Cagney et al., 2009). These findings highlight the strength of weak ties (Granovetter, 1973), consistent with the prior evidence that social connections at the more peripheral levels are beneficial to our well-being, often beyond strong ties such as with family and close friends (Sandstrom & Dunn, 2014). For older adults, socially cohesive neighborhoods may translate into better aging outcomes through several psychosocial and instrumental pathways, including diffusion of health information, provision of emotional and practical support among neighbors, maintenance of healthy lifestyles through informal social control, and the enhancement of mutual respect and self-efficacy (Cramm et al., 2013; Kawachi & Berkman, 2000).

Another finding deserving attention is the age difference, such that socially cohesive neighborhoods are associated with positive perceptions of aging more strongly for middle-aged residents. Socioemotional Selectivity Theory (Carstensen et al., 1999) provides a theoretical lens for interpretation. According to the theory, people place greater emphasis on social relationships with close partners at older ages and develop better regulation and greater control over emotions, whereas younger adults also focus on interacting with others from a broader social network such as neighbors (Carstensen et al., 1999). As the future time perspective becomes shorter, as it typically does with age, people tend to be increasingly selective and invest more resources in emotionally meaningful goals and activities than in knowledge acquisition. Although the age differences are most pronounced between early adulthood (e.g., the 20s and 30s) and older ages (e.g., the 80s), the theory conceptualizes middle ages as different from older ages, when the social motive for emotional regulation is still lower with the higher motive for knowledge acquisition compared to older ages (Carstensen et al., 1997). In this sense, distal social networks such as neighbors and a sense of belonging to local areas may have closer associations with middle-aged adults’ evaluations of their aging experiences. In contrast, older adults are likely to put more value and meaning in their interactions with family and close friends than in their connections to neighbors. Similarly, one recent study found that community cohesion was a significant predictor of loneliness for younger people aged 18–44, whereas family support mattered more for people aged 65 and above (Park et al., 2021). Taken together, these findings point out the importance of considering developmental stages and corresponding changes in social motives in relation to adults’ perceptions of growing older.

The strength of this study includes the use of a large, nationally representative sample of older Americans along with the neighborhood information drawn from the U.S. Census, allowing a more reliable interpretation at the national level. Moreover, we considered objective socioeconomic conditions of neighborhoods as well as subjectively perceived neighborhood characteristics. This approach allows us to test the significant associations between each neighborhood dimension and SPA, independently of one another. Finally, the integration of perspectives from ecology, environmental psychology, and gerontology contributes to understanding how the external environment relates to our evaluations of age-related changes from a multidisciplinary point of view.

Our study can make a key contribution to the literature on attitudes toward aging. Prior studies have primarily focused on identifying individual-level characteristics as predictors of older adults’ SPA (Marques et al., 2020). However, the current study went beyond by suggesting the significant role of a contextual-level factor. Not only that, but our findings might also extend the existing conceptual framework for subjective aging research. For example, Diehl et al. (2021) proposed an interdisciplinary model of awareness of aging-related change, which highlights that one’s perceptions related to own aging are influenced by a variety of distal (e.g., sociodemographic and health status) and proximal (e.g., personal goals and felt age) factors within a given cultural–societal context. A neighborhood social environment can serve as such an important cultural–societal context where SPA develops, as well as potentially bridge the broader context (e.g., cultural individualism or age-friendly society) to individuals’ evaluation processes of aging.

Our results suggest that community-level interventions aimed at promoting social cohesion within neighborhoods may help to increase more positive perceptions of aging. As reported by Higgins and Hunt (2016), individuals who use neighborhood facilities, volunteer, and attend community meetings are more likely to perceive their neighborhoods as socially cohesive. Thus, strategies such as creating age-friendly environments that promote social engagement and accessibility for older adults, as well as designing community programs to encourage community participation and volunteerism among older adults, can be implemented at the community level. These programs may include arts and activities, which have been found to be effective for residents’ active participation in community cultural activities (Jeannotte, 2003). Another potential avenue for improving social cohesion is to provide accessible and safe outdoor spaces and community recreation centers, which can offer more opportunities for building relationships with neighbors (Breedvelt et al., 2022). Furthermore, efforts may also aim to create combined community interventions of education and intergenerational contact, which are known to be the most effective intervention to reduce ageism (Burnes et al., 2019), to facilitate social connections between older and younger residents.

There are a few limitations worth noting. First, the present study used cross-sectional data, which does not allow claiming any causal argument. Future longitudinal research design would provide better insights into the temporal ordering of the relationship between neighborhood social environment and SPA scores while addressing concerns about selection or omitted variable bias. Particularly, future research can test the bidirectional association between neighborhood social cohesion and SPA. It is plausible that individuals with more positive SPA may be more likely to engage with their communities and thus report their neighborhoods to be more socially cohesive. Such investigation would provide a clearer understanding of how neighborhood environments shape one’s perceptions of aging. Second, our measure of the proportion of older adults in the neighborhood included individuals in long-term care facilities. This may result in some neighborhoods being categorized as “super-aged” simply due to the presence of a long-term care facility, rather than a high concentration of older adults in the community. This could affect the comparability of older adults living in these neighborhoods versus those living in communities without long-term care facilities, potentially confounding the relationship between the neighborhood indicator and SPA. Although our data did not allow us to test this hypothesis, future research is needed to explore whether the presence of long-term care facilities may affect older adults’ perceptions of aging. Additionally, although this study used large, nationally representative data, the sample largely consisted of non-Hispanic Whites. Future studies should include more people from diverse racial/ethnic backgrounds and explore whether the findings remain similar or vary. Finally, this is an exploratory study and given there is limited research in this area, we chose indicators of neighborhood social environment that have been used in prior studies (Wolff et al., 2018; Wurm et al., 2014) and have been shown to be significant predictors of general forms of ageism (Marques et al., 2020). It is important to note that these indicators may not fully capture the complexity of the neighborhood social environment. Further conceptual development is necessary to provide a more systematic selection of indicators and to identify other potentially relevant indicators.

## Conclusion

Grounded in the Ecology of Human Development framework and the Ecological Model of Aging, this study identified significant indicators of neighborhood social environment in predicting SPA among a nationally representative sample of adults aged 50 and above. These results provide some of the first insights into how neighborhood social context relates to one’s perceptions of own aging, suggesting that a socially cohesive neighborhood may be critical to promoting more favorable perceptions of aging, particularly in middle age. In addition to the prior focus on individual-level interventions, our study emphasizes the need for community intervention strategies to bolster the cohesion of neighborhoods, which can translate into better self-perceptions of aging among older residents.

## Data Availability

Data used in this study are publicly available and restricted data sets from the Health and Retirement Study (HRS), which is sponsored by the National Institute on Aging (NIA U01AG009740) and conducted by the University of Michigan website. The analytic data sets for this study incorporate restricted data sources from the HRS. Due to privacy and confidentiality requirements, these datasets are not publicly available. The current research was not preregistered in an independent, institutional registry.

## References

[CIT0001] Ailshire, J., Mawhorter, S., & Choi, E. Y. (2020). Contextual Data Resource (CDR): US Decennial Census and American Community Survey Data, 1990–2018, Version 2.0. USC/UCLA Center on Biodemography and Population Health. https://hrs.isr.umich.edu/sites/default/files/restricted_data_docs/Census_ACS_HRS-CDR_Documentation_2020-09-14.pdf

[CIT0002] Barkoff, A. (2021, May 27). Just in time for older Americans month: 2020 profile of older Americans. Administration for Community Living. https://acl.gov/news-and-events/acl-blog/just-time-older-americans-month-2020-profile-older-americans

[CIT0003] Boardman, J. D., & Robert, S. A. (2000). Neighborhood socioeconomic status and perceptions of self-efficacy. Sociological Perspectives, 43(1), 117–136. doi:10.2307/1389785

[CIT0004] Bonnes, M., Lee, T., & Bonaiuto, M. (2003). Theory and practice in environmental psychology—An introduction. In M. Bonnes, T. Lee, & M. Bonaiuto (Eds.), Psychological theories for environmental issues (pp. 1–25). Routledge.

[CIT0005] Breedvelt, J. J. F., Tiemeier, H., Sharples, E., Galea, S., Niedzwiedz, C., Elliott, I., & Bockting, C. L. (2022). The effects of neighbourhood social cohesion on preventing depression and anxiety among adolescents and young adults: Rapid review. BJPsych Open, 8(4), e97. doi:10.1192/bjo.2022.57 35642359 PMC9230698

[CIT0006] Bronfenbrenner, U. (1977). Toward an experimental ecology of human development. American Psychologist, 32(7), 513–531. doi:10.1037/0003-066x.32.7.513

[CIT0007] Burnes, D., Sheppard, C., Henderson, C. R., Wassel, M., Cope, R., Barber, C., & Pillemer, K. (2019). Interventions to reduce ­ageism against older adults: A systematic review and meta-analysis. American Journal of Public Health, 109(8), e1–e9. doi:10.2105/AJPH.2019.305123 PMC6611108 31219720

[CIT0008] Cagney, K. A., Glass, T. A., Skarupski, K. A., Barnes, L. L., Schwartz, B. S., & Mendes de Leon, C. F. (2009). Neighborhood-level cohesion and disorder: Measurement and validation in two older adult urban populations. The Journals of Gerontology, Series B: Psychological Sciences and Social Sciences, 64B(3), 415–424. doi:10.1093/geronb/gbn041 PMC2670251 19255089

[CIT0009] Carstensen, L. L., Gross, J. J., & Fung, H. H. (1997). The social context of emotional experience. Annual Review of Gerontology and Geriatrics, 17(1), 325–352. doi:10.1891/0198-8794.17.1.325

[CIT0010] Carstensen, L. L., Isaacowitz, D. M., & Charles, S. T. (1999). Taking time seriously: A theory of socioemotional selectivity. American Psychologist, 54(3), 165–181. doi:10.1037/0003-066x.54.3.165 10199217

[CIT0011] Chang, E.-S., Kannoth, S., Levy, S., Wang, S.-Y., Lee, J. E., & Levy, B. R. (2020). Global reach of ageism on older persons’ health: A systematic review. PLoS One, 15(1), e0220857. doi:10.1371/journal.pone.0220857 31940338 PMC6961830

[CIT0012] Chopik, W. J., Bremner, R. H., Johnson, D. J., & Giasson, H. L. (2018). Age differences in age perceptions and developmental transitions. Frontiers in Psychology, 9, 67. doi:10.3389/fpsyg.2018.00067 29449823 PMC5799826

[CIT0013] Cornwell, B., Laumann, E. O., & Schumm, L. P. (2008). The social connectedness of older adults: A national profile. American Sociological Review, 73(2), 185–203. doi:10.1177/000312240807300201 19018292 PMC2583428

[CIT0014] Cramm, J. M., & Nieboer, A. P. (2015). Social cohesion and belonging predict the well-being of community-dwelling older people. BMC Geriatrics, 15(1), 30. doi:10.1186/s12877-015-0027-y 25879773 PMC4369354

[CIT0015] Cramm, J. M., van Dijk, H. M., & Nieboer, A. P. (2013). The importance of neighborhood social cohesion and social capital for the well being of older adults in the community. Gerontologist, 53(1), 142–152. doi:10.1093/geront/gns052 22547088

[CIT0016] Crane, J. (1991). The epidemic theory of Ghettos and neighborhood effects on dropping out and teenage childbearing. American Journal of Sociology, 96(5), 1226–1259. doi:10.1086/229654. https://www.jstor.org/stable/2781341

[CIT0017] Diehl, M., Brothers, A. F., & Wahl, H.-W.(2021). Chapter 10 - Self-perceptions and awareness of aging: Past, present, and future. In K. W. Schaie & S. L. Willis (Eds.), Handbook of the psychology of aging (9th ed.) (pp. 155–179). Elsevier. doi:10.1016/B978-0-12-816094-7.00001-5

[CIT0018] Diehl, M., Wahl, H.-W., Brothers, A., & Miche, M. (2015). Subjective aging and awareness of aging: Toward a new understanding of the aging self. In Annual review of gerontology and geriatrics, Vol. 35, 2015: Subjective aging: New developments and future directions (pp. 1–28). Springer Publishing Company.

[CIT0019] Fagg, J. H., Curtis, S. E., Cummins, S., Stansfeld, S. A., & Quesnel-Vallée, A. (2013). Neighbourhood deprivation and adolescent self-esteem: Exploration of the “socio-economic equalisation in youth” hypothesis in Britain and Canada. Social Science & Medicine, 91, 168–177. doi:10.1016/j.socscimed.2013.02.021 23518228 PMC3726937

[CIT0020] Granovetter, M. S. (1973). The strength of weak ties. American Journal of Sociology, 78(6), 1360–1380. doi:10.1086/225469. https://www.jstor.org/stable/2776392

[CIT0021] Hagestad, G. O., & Uhlenerg, P. (2005). The social separation of old and young: A root of ageism. Journal of Social Issues, 61(2), 343–360. doi:10.1111/j.1540-4560.2005.00409.x

[CIT0022] Higgins, B. R., & Hunt, J. (2016). Collective efficacy: Taking action to improve neighborhoods. NIJ Journal, 277, 18–21. http://nij.gov/journals/277/Pages/collective-efficacy.aspx

[CIT0023] James, W. (1890). The principles of psychology, Vol. 1. Henry Holt and Co. doi:10.1037/10538-000

[CIT0024] Jeannotte, M. S. (2003). Singing alone? The contribution of cultural capital to social cohesion and sustainable communities. International Journal of Cultural Policy, 9(1), 35–49. doi:10.1080/1028663032000089507

[CIT0025] Kawachi, I., & Berkman, L. F. (2000). Social capital, social cohesion, and health. In L. F. Berkman, I. Kawachi, & M. M. Glymour (Eds.), Social epidemiology (2nd ed.). Oxford University Press. https://oxfordmedicine.com/view/10.1093/med/9780195377903.001.0001/med-9780195377903-chapter-8

[CIT0026] Kornadt, A. E., Albert, I., Hoffmann, M., Murdock, E., & Nell, J. (2021 ). Perceived ageism during the Covid-19-crisis is longitudinally related to subjective perceptions of aging. Frontiers in Public Health, 9, 679711. doi:10.3389/fpubh.2021.679711 34327186 PMC8313802

[CIT0027] Kotter-Grühn, D., Kleinspehn-Ammerlahn, A., Gerstorf, D., & Smith, J. (2009). Self-perceptions of aging predict mortality and change with approaching death: 16-Year longitudinal results from the Berlin Aging Study. Psychology and Aging, 24(3), 654–667. doi:10.1037/a0016510 19739922

[CIT0028] Kubzansky, L. D., Subramanian, S. V., Kawachi, I., Fay, M. E., Soobader, M.-J., & Berkman, L. F. (2005). Neighborhood contextual influences on depressive symptoms in the elderly. American Journal of Epidemiology, 162(3), 253–260. doi:10.1093/aje/kwi185 15987730

[CIT0029] Lawton, M. P. (1975). The Philadelphia Geriatric Center Morale Scale: A revision. Journal of Gerontology, 30(1), 85–89. doi:10.1093/geronj/30.1.85 1109399

[CIT0030] Lawton, M. P., & Nahemow, L. (1973). Ecology and the aging process. In C. Eisdorfer & M. P. Lawton (Eds.), The psychology of adult development and aging (pp. 619–674). American Psychological Association. doi:10.1037/10044-020

[CIT0031] Levy, B. R. (2003). Mind matters: Cognitive and physical effects of aging self-stereotypes. The Journals of Gerontology, Series B: Psychological Sciences and Social Sciences, 58(4), P203–P211. doi:10.1093/geronb/58.4.p203 12878645

[CIT0032] Marques, S., Mariano, J., Mendonça, J., De Tavernier, W., Hess, M., Naegele, L., Peixeiro, F., & Martins, D. (2020). Determinants of ageism against older adults: A systematic review. International Journal of Environmental Research and Public Health, 17(7), 2560. doi:10.3390/ijerph17072560 32276489 PMC7178234

[CIT0033] Mead, G. H. (1934). Mind, self, and society. University of Chicago Press.

[CIT0034] Miche, M., Wahl, H.-W., Diehl, M., Oswald, F., Kaspar, R., & Kolb, M. (2014). Natural occurrence of subjective aging experiences in community-dwelling older adults. The Journals of Gerontology, Series B: Psychological Sciences and Social Sciences, 69(2), 174–87. doi:10.1093/geronb/gbs164 23325506 PMC12124237

[CIT0035] Neugarten, B. L. (1975). The future and the young-old. Gerontologist, 15(1 Part 2), 4–9. doi:10.1093/geront/15.1_part_2.4 1110022

[CIT0036] Nordstrom, C. K., Diez Roux, A. V., Jackson, S. A., & Gardin, J. M. (2004). The association of personal and neighborhood socioeconomic indicators with subclinical cardiovascular disease in an ­elderly cohort. The Cardiovascular Health Study. Social Science & Medicine, 59(10), 2139–2147. doi:10.1016/j.socscimed.2004.03.017 15351479

[CIT0037] OECD & World Health Organization. (2020). Health at a glance: Asia/Pacific 2020: Measuring progress towards universal health coverage. OECD. doi:10.1787/26b007cd-en

[CIT0038] Oswald, F., & Wahl, H. (2005). Dimensions of the meaning of home in later life. In G. D. Rowles & H. Chaudhury (Eds.), Home and identity in later life: International perspectives (pp. 21–45). Springer.

[CIT0039] Park, N. S., Chiriboga, D. A., & Chung, S. (2021). The effect of social capital and family support on loneliness among Korean adults: Intergenerational differences. Journal of Intergenerational Relationships, 19(1), 109–123. doi:10.1080/15350770.2021.1868239

[CIT0040] Pretty, G. H., Chipuer, H. M., & Bramston, P. (2003). Sense of place amongst adolescents and adults in two rural Australian towns: The discriminating features of place attachment, sense of community and place dependence in relation to place identity. Journal of Environmental Psychology, 23(3), 273–287. doi:10.1016/s0272-4944(02)00079-8

[CIT0041] Proshansky, H. M. (1978). The city and self-identity. Environment and Behavior, 10(2), 147–169. doi:10.1177/0013916578102002

[CIT0042] Radloff, L. S. (1977). The CES-D Scale: A self-report depression scale for research in the general population. Applied Psychological Measurement, 1(3), 385–401. doi:10.1177/014662167700100306

[CIT0043] Sandstrom, G. M., & Dunn, E. W. (2014). Social interactions and well-being: The surprising power of weak ties. Personality & Social Psychology Bulletin, 40(7), 910–922. doi:10.1177/0146167214529799 24769739

[CIT0044] Skerrett, K., Spira, M., & Chandy, J. (2021). Emerging elderhood: Transitions from midlife. Clinical Social Work Journal, 50(4), 377–386. doi:10.1007/s10615-021-00791-2 33612873 PMC7884206

[CIT0045] Smith, J., Ryan, L., Fisher, G. G., Sonnega, A., & Weir, D. (2017). Psychosocial and lifestyle questionnaire 2006—2016. Survey ­Research Center Institute for Social Research University of ­Michigan. https://hrs.isr.umich.edu/sites/default/files/biblio/HRS%202006-2016%20SAQ%20Documentation_07.06.17_0.pdf

[CIT0046] Stokes, J. E., & Moorman, S. M. (2016). Who are the people in your neighborhood? Neighborhood age composition and age discrimination. Social Psychology Quarterly, 79(1), 68–80. doi:10.1177/0190272515626569

[CIT0047] Subramanian, S. V., Kubzansky, L., Berkman, L., Fay, M., & Kawachi, I. (2006). Neighborhood effects on the self-rated health of elders: Uncovering the relative importance of structural and service-related neighborhood environments. The Journals of Gerontology, Series B: Psychological Sciences and Social Sciences, 61(3), S153–S160. doi:10.1093/geronb/61.3.S153 16670193

[CIT0048] Tully-Wilson, C., Bojack, R., Millear, P. M., Stallman, H. M., Allen, A., & Mason, J. (2021). Self-perceptions of aging: A systematic review of longitudinal studies. Psychology and Aging, 36, 773–789. doi:10.1037/pag0000638 34498894

[CIT0049] Wahl, H.-W. (2015). Theories of environmental influences on aging and behavior. In N. A. Pachana (Ed.), Encyclopedia of geropsychology (pp. 1–8). Springer Singapore. doi:10.1007/978-981-287-080-3_132-1

[CIT0050] Wahl, H.-W., Iwarsson, S., & Oswald, F. (2012). aging well and the environment: Toward an integrative model and research agenda for the future. Gerontologist, 52(3), 306–316. doi:10.1093/geront/gnr154 22419248

[CIT0051] Wolff, J. K., Beyer, A.-K., Wurm, S., Nowossadeck, S., & Wiest, M. (2018). Regional impact of population aging on changes in individual self-perceptions of aging: Findings from the German Ageing Survey. Gerontologist, 58(1), 47–56. doi:10.1093/geront/gnx127 28958001

[CIT0052] Wurm, S., Wolff, J. K., & Schüz, B. (2014). Primary care supply moderates the impact of diseases on self-perceptions of aging. Psychology and Aging, 29(2), 351–358. doi:10.1037/a0036248 24956003

[CIT0053] Yen, I. H., Michael, Y. L., & Perdue, L. (2009). Neighborhood environment in studies of health of older adults. American Journal of Preventive Medicine, 37(5), 455–463. doi:10.1016/j.amepre.2009.06.022 19840702 PMC2785463

